# Supplementary material for: Analysis of PPARγ Signaling Activity in Psoriasis
Source: Int J Mol Sci. 2021 Aug 10;22(16):8603. doi: 10.3390/ijms22168603 (PMC8395241; doi:10.3390/ijms22168603)
Supplement: Supplementary file 1 [file ijms-22-08603-s001.zip › Supplemental materials_Analysis of PPARg signaling activity in psoriasis/Pathway models/Models images and html files/Interleukin-17 and interleukin-22 signaling in psoriasis/7534.html]

MMP9


# Protein MMP9

|  |  |
| --- | --- |
| URN | urn:agi-llid:4318 |
| Total Entities | 0 |
| Connectivity | 17216 |
| Name | MMP9 |
| Description | matrix metallopeptidase 9 (gelatinase B, 92kDa gelatinase, 92kDa type IV collagenase) |
| Notes | Proteins of the matrix metalloproteinase (MMP) family are involved in the breakdown of extracellular matrix in normal physiological processes, such as embryonic development, reproduction, and tissue remodeling, as well as in disease processes, such as arthritis and metastasis. Most MMP's are secreted as inactive proproteins which are activated when cleaved by extracellular proteinases. The enzyme encoded by this gene degrades type IV and V collagens. Studies in rhesus monkeys suggest that the enzyme is involved in IL-8-induced mobilization of hematopoietic progenitor cells from bone marrow, and murine studies suggest a role in tumor-associated tissue remodeling. [provided by RefSeq, Jul 2008] |
| Primary Cell Localization | Extracellular |
| ObjectType | Protein |

---

|  |  |
| --- | --- |
| Pathway | Alzheimer's Disease |
|  | Skin Aging |
|  | Proplatelet Maturation |
|  | Lipoxin A4/FPR2-Related Neutrophil Depression |
|  | Neutrophil Activation via Adherence on Endothelial Cells |
|  | Nociception Expression Targets Signaling |
|  | CCR1 Expression Targets |
|  | Extracellular Matrix Turnover |
|  | Proteins Involved in Atherosclerosis |
|  | Dilated Cardiomyopathy |
|  | Proteins Involved in Dilated Cardiomyopathy |
|  | Cardiomyocyte Hypertrophy |
|  | Hypertrophic Cardiomyopathy |
|  | Proteins Involved in Arterial Hypertension |
|  | Proteins Involved in Pulmonary Hypertension |
|  | Smooth Muscle Cell Dysfunction in Pulmonary Hypertension |
|  | Myocardial Remodeling |
|  | Myocardial Infarction |
|  | Proteins Involved in Myocardial Ischemia |
|  | Proteins Involved in Myocarditis |
|  | Inflammatory Reaction in Acne Vulgaris |
|  | Proteins with Altered Expression in Melanoma |
|  | Melanoma |
|  | Proteins Involved in Melanoma |
|  | IL17 Signaling in Psoriasis |
|  | Proteins Involved in Psoriasis |
|  | IL22 Induces Keratinocyte Proliferation in Psoriasis |
|  | Proteins with Altered Expression in Psoriasis |
|  | Proteins Involved in Photoaging |
|  | Extracellular Matrix Degradation in Skin Aging |
|  | Proteins Involved in Systemic Lupus Erythematosus |
|  | Tubular Cell Dysfunction in Progressive Diabetic Nephropathy |
|  | Proteins Involved in Diabetic Nephropathy |
|  | Mesangial Cell Dysfunction in Diabetic Nephropathy |
|  | Hepatic Stellate Cells in non-Alcoholic Fatty Liver Disease |
|  | Proteins Involved in Colorectal Neoplasms |
|  | Metastatic Colorectal Cancer |
|  | Proteins Involved in Ulcerative Colitis |
|  | Proteins Involved in B-Cell Chronic Lymphocytic Leukemia |
|  | B-Cell Chronic Lymphocytic Leukemia |
|  | Osteoclast Activation in Multiple Myeloma |
|  | IL6/IGF1/VEGFA Signaling in Multiple Myeloma |
|  | Proteins Involved in Multiple Myeloma |
|  | Proteins Involved in Hepatocellular Carcinoma |
|  | Proteins Involved in Pancreatic Cancer |
|  | Neutrophil Degranulation via FPR1/IL8 |
|  | Proteins Involved in Osteoarthritis |
|  | Osteoclast Activation in Psoriatic Arthritis |
|  | Synovial Fibroblast Activation in Psoriatic Arthritis |
|  | Keratinocyte Activation in Psoriatic Arthritis |
|  | Proteins Involved in Rheumatoid Arthritis |
|  | Proteins Involved in Systemic Scleroderma |
|  | Skin Fibrosis |
|  | Cathepsins Role in Periodontitis |
|  | Estrogen Effects on Pregnancy Gingivitis |
|  | Proteins Involved in Periodontitis |
|  | Proteins Involved in Fragile X Syndrome |
|  | Muscular Dystrophies |
|  | Proteins Involved in Alzheimer's Disease |
|  | Amyloid beta Traffic and Degradation in Extracellular Matrix in Alzheimer's Disease |
|  | Proteins Involved in Epilepsy |
|  | Proteins Involved in Glioblastoma |
|  | Glioma Invasion Signaling |
|  | Proteins Involved in Glioma |
|  | Proteins Involved in Medulloblastoma |
|  | Proteins Involved in Neuroblastoma |
|  | Proteins Involved in Amyotrophic Lateral Sclerosis |
|  | Proteins Involved in Multiple Sclerosis |
|  | Proteins Involved in Breast Cancer Related to WNT Signaling Pathway |
|  | Proteins Involved in Breast Cancer Related to ESR1 Signaling Pathway |
|  | Proteins Involved in Breast Cancer Related to NOTCH Signaling Pathway |
|  | WNT Signaling in Breast Cancer |
|  | Proteins Involved in Breast Cancer Related to ERBB2/VEGFR/Akt Signaling Pathway |
|  | Proteins with Altered Expression in Breast Cancer |
|  | Proteins Involved in Breast Cancer Related to IGF1R/Akt Signaling Pathway |
|  | NOTCH1 Signaling in Breast Cancer |
|  | Proteins Involved in Adhesion, Migration and Invasion of Endometriotic Cell |
|  | Proteins Involved in Endometriosis |
|  | Proteins with Altered Expression in Endometriosis |
|  | Proteins Involved in Cholesteatoma |
|  | MIF Role in Cholesteatoma |
|  | Cholesteatoma |
|  | Prostate Cancer |
|  | Proteins Involved in Prostate Cancer |
|  | Netrin-1 Signaling in Age-Related Macular Degeneration |
|  | Proteins Involved in Age-Related Macular Degeneration |
|  | Proteins Involved in Cataract |
|  | Proteins with Altered Expression in Cataract |
|  | Steroids Induced Cataract |
|  | Proteins Involved in Glaucoma |
|  | PGF2-alpha Effect on Treatment |
|  | Proteins Involved in Otitis Media |
|  | Proteins with Altered Expression in Asthma |
|  | Alveolar Macrophages Dysfunction in COPD |
|  | Proteins Involved in Chronic Obstructive Pulmonary Disease |
|  | Neutrophil Associated Lung Damage in Chronic Obstructive Pulmonary Disease |
|  | Proteins Involved in Glomerulonephritis |
|  | Mesangial Cell Dysfunction in Glomerulonephritis |
|  | Interstitial Fibroblasts in Pyelonephritis |
|  | HMGB1 Expression Targets |
|  | ANGPT2 -> AP-1/CREBBP/MYC Expression Targets |
|  | AREG -> CTNN Expression Targets |
|  | AREG -> STAT Expression Targets |
|  | AREG -> AP-1 Expression Targets |
|  | Noradrenaline/Gs Expression Targets |
|  | Noreadrenaline/Gq Expression Targets |
|  | Acetylcholine Expression Targets |
|  | BMP6/ACVR2A Expression Targets |
|  | BTC -> NFATC Expression Targets |
|  | BTC -> AP-1/ATF/CREB Expression Targets |
|  | BTC -> CTNN Expression Targets |
|  | BTC -> STAT Expression Targets |
|  | BTC -> EP300/ETS/ETV/SP1 Expression Targets |
|  | Elevated Receptors -> Expression Targets in Joint |
|  | Receptor Tyrosine Kinase Family -> Expression Targets in Bone |
|  | GPCRs Family -> Expression Targets in Bone |
|  | Elevated Receptors -> Expression Targets in Bone |
|  | CD86 -> NFATC Expression Targets |
|  | Elevated Receptors -> Expression Targets in Blood vessel |
|  | Elevated Receptors -> Expression Targets in Blood Vessel |
|  | CXCL1 Expression Targets |
|  | CXCL12 Expression Targets |
|  | CCL3 Expression Targets |
|  | CCL7 Expression Target |
|  | CCL7 Expression Targets |
|  | CCL8 Expression Targets |
|  | IL8 Expression Targets |
|  | CSF3 Expression Targets |
|  | CSF2 -> STAT Expression Targets |
|  | EGF -> CTNN Expression Targets |
|  | EGF -> AP-1/ATF Expression Targets |
|  | EGF -> CREB/CREBBP/ELK/SRF/MYC Expression Targets |
|  | EGF -> MEF/MYOD/NFATC Expression Targets |
|  | EGF -> STAT Expression Targets |
|  | Erythropoietin -> NF-kB Expression Targets |
|  | Erythropoietin -> AP-1/MYC/CREB Expression Targets |
|  | Erythropoietin -> STAT Expression Targets |
|  | Erythropoietin -> ELK/SRF Expression Targets |
|  | F2 -> AP-1/CREB/ELK/SRF/SP1 Expression Targets |
|  | F2 -> STAT1/NF-kB Expression Targets |
|  | FGF2 -> STAT Expression Targets |
|  | FGF1 -> AP-1/CREB/ELK/SRF/MYC Expression Targets |
|  | FGF8 -> AP-1/CREB/MYC Expression Targets |
|  | FGF1 -> STAT Expression Targets |
|  | FGF8 -> STAT Expression Targets |
|  | FGF8 -> RUNX Expression Targets |
|  | FGF2 -> AP-1/CREB/CREBBP/ELK/SRF/MYC Expression Targets |
|  | ADCYAP1 Expression Targets |
|  | Elevated Receptors -> Expression Targets in Liver |
|  | HGF -> STAT Expression Targets |
|  | HGF -> AP-1/CREB/ELK/SRF/MYC Expression Targets |
|  | EGFR Transactivation in Cancer and Non-Cancer Cells |
|  | TAM Receptor Signaling Activation in Cancer |
|  | STAT3 and NFkB Activate Inflammation-Induced Tumorigenesis |
|  | IL13/STAT6/ZEB1 Signalling Promotes Cancer Metastatic Progression |
|  | N2 Neutrophils in Tumor-Promoting Inflammation and Tumor Progression |
|  | Oxidized LDL/OLR1 Activates Cell Proliferation in Cancer |
|  | Inhibition of THBS1 Activates Angiogenesis in Cancer |
|  | Integrins in Cancer Cell Motility, Invasion and Survival |
|  | Proteins with Altered Expression in Cancer Metastases |
|  | Epithelial to Mesenchymal Transition in Cancer: Overview |
|  | BSG (CD147) in Cancer Cells Motility, Invasion and Survival |
|  | MTOR/TORC in Cancer Cell Motility and Invasion |
|  | TGFB Family in Epithelial to Mesenchymal Transition in Cancer |
|  | Invadopodia Formation in Cancer Cells |
|  | WNT in Epithelial to Mesenchymal Transition in Cancer |
|  | Tumor Infiltrating Macrophages in Cancer Progression and Immune Escape |
|  | INHBA/ACVR2/BMPR Expression Targets |
|  | INHBA/ACVR2/ACVR1 Expression Targets |
|  | Insulin -> STAT Expression Targets |
|  | Insulin -> CEBPA/CTNNB/FOXA/FOXO Expression Targets |
|  | Insulin -> MEF/MYOD Expression Targets |
|  | Insulin -> ELK/SRF/HIF1A/MYC/SREBF Expression Targets |
|  | Fibronectin Expression Targets |
|  | CTGF -> AP-1/CREB/MYC Expression Targets |
|  | ICAM1 Expression Targets |
|  | EGFR -> Expression Targets in Skin |
|  | Elevated Receptors -> Expression Targets in Skin |
|  | IFNG/IFNR Expression Targets |
|  | IL13 Expression Targets |
|  | IL21 Expression Targets |
|  | IL6 Expression Targets |
|  | IL11 Expression Targets |
|  | IL4 Expression Targets |
|  | IL2 Expression Targets |
|  | Leptin -> STAT Expression Targets |
|  | Leptin -> ELK/SRF Expression Targets |
|  | Prostaglandin F Expression Targets |
|  | PAF/Gq -> AP-1/ATF1/CREB/ERK/SRF Expression Targets |
|  | S1P Expression Targets |
|  | PGE1 Expression Targets |
|  | EGFR-> Expression Targets in Lymphoid System and Blood |
|  | Elevated Receptors -> Expression Targets in Lymph Node |
|  | Elevated Receptors -> Expression Targets in Lymphoid System and Blood |
|  | F2R/F2RL1 -> Expression Targets in Lymphoid System and Blood |
|  | CD Markers -> Expression Targets in Lymphoid System and Blood |
|  | GPCRs Family -> Expression Targets in Lymphoid System and Blood |
|  | Cell Adhesion Receptors -> Expression Targets in Lymphoid System and Blood |
|  | Immunoglobulin Superfamily -> Expression Targets in Lymphoid System and Blood |
|  | Elevated Receptors -> Expression Targets in Bone Marrow |
|  | Interleukin Receptors -> Expression Targets in Lymphoid System and Blood |
|  | C5AR1 -> Expression Targets in Lymphoid System and Blood |
|  | Chemokine Receptor Family -> Expression Targets in Lymphoid System |
|  | NRG1 -> EP300/ETS/ETV/SP1 Expression Targets |
|  | NRG1 -> CREB/CREBBP/ELK/SRF/MYC Expression Targets |
|  | NRG1/Catenin Expression Targets |
|  | NRG1 -> STAT Expression Targets |
|  | NRG1 -> AP-1/ATF Expression Targets |
|  | Elevated Receptors -> Expression Targets in Nerve Tissue |
|  | NGF -> SMAD3/NF-kB Expression Targets |
|  | NGF -> AP-1/TP53/MYC Expression Targets |
|  | NOTCH Expression Targets |
|  | PDGFD -> AP-1 Expression Targets |
|  | PDGFD -> STAT Expression Targets |
|  | PDGF -> AP-1/CREB/CREBBP/MYC Expression Targets |
|  | PDGF -> STAT Expression Targets |
|  | GNRH1 Expression Targets |
|  | NTS Expression Targets |
|  | GAST Expression Targets in GAST Expression Targets |
|  | EDN1 Expression Targets |
|  | AGT -> CREB Expression Targets |
|  | AGT -> STAT Expression Targets |
|  | AVP/Gq -> CREB/ELK/SRF/AP-1/EGR Expression Targets |
|  | PRL/GHR -> STAT Expression Targets |
|  | PRL/GHR -> NF/kB/ELK/SRF/MYC Expression Targets |
|  | KITLG -> STAT Expression Targets |
|  | KITLG -> AP-1/CREB/CREBBP/MYC Expression Targets |
|  | EphrinR Expression Targets |
|  | GDNF -> HSF1 Expression Targets |
|  | PAF Expression Targets |
|  | PGF -> AP-1/CREB/CREBBP/MYC Expression Targets |
|  | Elevated Receptors -> Expression Targets in Breast |
|  | Elevated Receptors -> Expression Targets in Uterus |
|  | Elevated Receptors -> Expression Targets in Lung |
|  | TGFA -> STAT Expression Targets |
|  | TGFA -> CTNNB/CTNND Expression Targets |
|  | TGFA/AP-1/ATF Expression Targets |
|  | TGFA -> CREB/CREBBP/ELK-SRF/MYC Expression Targets |
|  | TGFB3-TGFBR1 Expression Targets |
|  | TGFB1-TGFBR1 Expression Targets |
|  | TGFB3-TGFBR2 Expression Targets |
|  | TGFB1-TGFBR2 Expression Targets |
|  | TGFB1-TGFBR1/AP-1 Expression Targets |
|  | TGFB1-ACVRL1 Expression Targets |
|  | TNF -> NF-kB Expression Targets |
|  | TNFSF10 Expression Targets |
|  | LTA Expression Targets |
|  | TNF -> AP-1 Expression Targets |
|  | CD72 -> AP-1 Expression Targets |
|  | CD40LG -> STAT Expression Targets |
|  | CD40LG -> NF-kB/ELK/SRF -> CREB/NFATC Expression Targets |
|  | TNF -> STAT Expression Targets |
|  | TLR3 Expression Targets |
|  | TLR4 -> AP-1 Expression Targets |
|  | TLR5 Expression Targets |
|  | IL1B -> NO Expression Targets |
|  | IL1B -> PGE2 Expression Targets |
|  | IL1B Expression Targets |
|  | IL1A Expression Targets |
|  | TLR9 Expression Targets |
|  | ROS in Triggering Vascular Inflammation |
|  | ROS in Angiotensin Mediated Cardiovascular Remodeling and Hypertrophy |
|  | PLAU -> STAT1 Expression Targets |
|  | PLAU -> ELK-SRF/AP-1 Expression Targets |
|  | VEGFA -> ATF/CREB/ELK-SRF Expression Targets |
|  | VEGFA -> STAT Expression Targets |
|  | VEGFA -> AP-1/CREBBP/MYC Expression Targets |
|  | VEGFA -> CTNNB/CTNND Expression Targets |
|  | WNT5A Expression Targets |
|  | WNT Canonical Signaling Expression Targets |
|  | pprarg neg, uknown targets, ps-positive |
|  | prarg neg,ukn expres targets, ps-positive |
|  | PPARG negative targets |
|  | pparg expr target\_regulators |
|  | Neighbors of disease exacerbation |
|  | Neighbors of tumor progression |
|  | Neighbors of cancer progression |
|  | Neighbors of inflammatory status |
|  | Neighbors of overall survival |
|  | Neighbors of disease severity |
|  | Neighbors of tumor size |
|  | Neighbors of lesion size |
|  | Neighbors of mortality |
|  | Neighbors of survival rate |
|  | Neighbors of infarct size |
|  | Neighbors of survival time |
|  | Neighbors of functional recovery |
|  | Neighbors of insulin sensitivity |
|  | Neighbors of bacterial load |
|  | Neighbors of cancer survival |
|  | Neighbors of graft survival |
|  | Neighbors of therapeutic efficacy |
|  | Neighbors of clinical stage |
|  | Neighbors of lethality |
|  | Neighbors of tumor regression |
|  | Neighbors of recurrence-free survival |
|  | Neighbors of disease-free survival |
|  | Neighbors of tumor response |
|  | Neighbors of macrophage count |
|  | Neighbors of treatment outcome |
|  | Neighbors of body weight gain |
|  | Neighbors of cardiovascular risk |
|  | Neighbors of microvessel density |
|  | Neighbors of tumor recurrence |
|  | Neighbors of tumor promotion |
|  | Neighbors of neutrophil count |
|  | Neighbors of morbidity |
|  | Neighbors of mortality rate |
|  | Neighbors of bone mineral density |
|  | Neighbors of adverse outcome |
|  | Neighbors of radioresistance |
|  | Neighbors of lesion area |
|  | Neighbors of cell density |
|  | Neighbors of cancer-specific survival |
|  | Neighbors of progression-free survival |
|  | Neighbors of shear stress |
|  | Neighbors of viral load |
|  | Neighbors of blood pressure |
|  | Neighbors of tumor grade |
|  | Neighbors of vascular reactivity |
|  | Neighbors of vascular leakage |
|  | Neighbors of body mass index |
|  | Neighbors of vascular density |
|  | Neighbors of cancer risk |
|  | Neighbors of cancer incidence |
|  | Neighbors of tumor aggressiveness |
|  | Neighbors of blood glucose |
|  | Neighbors of ejection fraction |
|  | Neighbors of renal hypertrophy |
|  | Neighbors of cancer recurrence |
|  | Neighbors of antimetastatic activity |
|  | Neighbors of glucose level |
|  | Neighbors of vascular tone |
|  | Neighbors of left ventricular ejection fraction |
|  | Neighbors of breast cancer risk |
|  | Neighbors of proliferation index |
|  | Neighbors of cell permeability |
|  | Neighbors of pregnancy outcome |
|  | Neighbors of functional status |
|  | Neighbors of neurological outcome |
|  | Neighbors of infarct area |
|  | Neighbors of fertility |
|  | Neighbors of recurrence rate |
|  | Neighbors of pathological response |
|  | Neighbors of skin thickness |
|  | Neighbors of carotid intima-media thickness |
|  | Neighbors of fibroblast phenotype |
|  | Neighbors of disease exacerbation |
|  | Neighbors of tumor progression |
|  | Neighbors of cancer progression |
|  | Neighbors of inflammatory status |
|  | Neighbors of overall survival |
|  | Neighbors of disease severity |
|  | Neighbors of tumor size |
|  | Neighbors of lesion size |
|  | Neighbors of mortality |
|  | Neighbors of survival rate |
|  | Neighbors of infarct size |
|  | Neighbors of survival time |
|  | Neighbors of functional recovery |
|  | Neighbors of insulin sensitivity |
|  | Neighbors of bacterial load |
|  | Neighbors of cancer survival |
|  | Neighbors of graft survival |
|  | Neighbors of therapeutic efficacy |
|  | Neighbors of clinical stage |
|  | Neighbors of lethality |
|  | Neighbors of tumor regression |
|  | Neighbors of recurrence-free survival |
|  | Neighbors of disease-free survival |
|  | Neighbors of tumor response |
|  | Neighbors of macrophage count |
|  | Neighbors of treatment outcome |
|  | Neighbors of body weight gain |
|  | Neighbors of cardiovascular risk |
|  | Neighbors of microvessel density |
|  | Neighbors of tumor recurrence |
|  | Neighbors of tumor promotion |
|  | Neighbors of neutrophil count |
|  | Neighbors of morbidity |
|  | Neighbors of mortality rate |
|  | Neighbors of bone mineral density |
|  | Neighbors of adverse outcome |
|  | Neighbors of radioresistance |
|  | Neighbors of lesion area |
|  | Neighbors of cell density |
|  | Neighbors of cancer-specific survival |
|  | Neighbors of progression-free survival |
|  | Neighbors of shear stress |
|  | Neighbors of viral load |
|  | Neighbors of blood pressure |
|  | Neighbors of tumor grade |
|  | Neighbors of vascular reactivity |
|  | Neighbors of vascular leakage |
|  | Neighbors of body mass index |
|  | Neighbors of vascular density |
|  | Neighbors of cancer risk |
|  | Neighbors of cancer incidence |
|  | Neighbors of tumor aggressiveness |
|  | Neighbors of blood glucose |
|  | Neighbors of ejection fraction |
|  | Neighbors of renal hypertrophy |
|  | Neighbors of cancer recurrence |
|  | Neighbors of antimetastatic activity |
|  | Neighbors of glucose level |
|  | Neighbors of vascular tone |
|  | Neighbors of left ventricular ejection fraction |
|  | Neighbors of breast cancer risk |
|  | Neighbors of proliferation index |
|  | Neighbors of cell permeability |
|  | Neighbors of pregnancy outcome |
|  | Neighbors of functional status |
|  | Neighbors of neurological outcome |
|  | Neighbors of infarct area |
|  | Neighbors of fertility |
|  | Neighbors of recurrence rate |
|  | Neighbors of pathological response |
|  | Neighbors of skin thickness |
|  | Neighbors of carotid intima-media thickness |
|  | Neighbors of fibroblast phenotype |
|  | Neighbors of cell development |
|  | Neighbors of immune response |
|  | Neighbors of cell population |
|  | Neighbors of inflammatory response |
|  | Neighbors of wound healing |
|  | Neighbors of angiogenesis |
|  | Neighbors of innate immune response |
|  | Neighbors of cell function |
|  | Neighbors of cell formation |
|  | Neighbors of adaptive immune response |
|  | Neighbors of osteoclast development |
|  | Neighbors of epithelial to mesenchymal transition |
|  | Neighbors of immunity |
|  | Neighbors of cell proliferative response |
|  | Neighbors of chemotaxis |
|  | Neighbors of dendritic cell differentiation |
|  | Neighbors of cell survival |
|  | Neighbors of osteoclast differentiation |
|  | Neighbors of cell phenotype |
|  | Neighbors of T-cell proliferation |
|  | Neighbors of tissue remodeling |
|  | Neighbors of cell adhesion |
|  | Neighbors of T-cell activation |
|  | Neighbors of endothelial cell proliferation |
|  | Neighbors of fibrogenesis |
|  | Neighbors of tumor growth |
|  | Neighbors of dendritic cell development |
|  | Neighbors of tissue repair |
|  | Neighbors of cell count |
|  | Neighbors of cell infiltration |
|  | Neighbors of cell interaction |
|  | Neighbors of SMC proliferation |
|  | Neighbors of lumen formation |
|  | Neighbors of endothelial cell migration |
|  | Neighbors of adipogenesis |
|  | Neighbors of lipid storage |
|  | Neighbors of cell damage |
|  | Neighbors of pregnancy |
|  | Neighbors of monocyte differentiation |
|  | Neighbors of aging |
|  | Neighbors of cell motility |
|  | Neighbors of bone resorption |
|  | Neighbors of monocyte migration |
|  | Neighbors of fibroblast proliferation |
|  | Neighbors of adipocyte differentiation |
|  | Neighbors of macrophage migration |
|  | Neighbors of microglial activation |
|  | Neighbors of neutrophil recruitment |
|  | Neighbors of regeneration |
|  | Neighbors of cell migration |
|  | Neighbors of cancer growth |
|  | Neighbors of stem cell proliferation |
|  | Neighbors of smooth muscle cell migration |
|  | Neighbors of hepatic regeneration |
|  | Neighbors of transendothelial migration |
|  | Neighbors of phagocytosis |
|  | Neighbors of leukocyte migration |
|  | Neighbors of leukocyte recruitment |
|  | Neighbors of osteoclast formation |
|  | Neighbors of monocyte recruitment |
|  | Neighbors of cell invasion |
|  | Neighbors of heart function |
|  | Neighbors of hemopoiesis |
|  | Neighbors of bone remodeling |
|  | Neighbors of ossification |
|  | Neighbors of immune system function |
|  | Neighbors of lung development |
|  | Neighbors of autophagy |
|  | Neighbors of monocyte adhesion |
|  | Neighbors of transcription activation |
|  | Neighbors of senescence |
|  | Neighbors of T-cell response |
|  | Neighbors of hepatocyte proliferation |
|  | Neighbors of cell transdifferentiation |
|  | Neighbors of neuroprotection |
|  | Neighbors of stem cell differentiation |
|  | Neighbors of endothelial cell function |
|  | Neighbors of neuronal death |
|  | Neighbors of cell growth |
|  | Neighbors of ROS generation |
|  | Neighbors of epithelial cell proliferation |
|  | Neighbors of first trimester pregnancy |
|  | Neighbors of cancer cell growth |
|  | Neighbors of sensitization |
|  | Neighbors of hemato-encephalic barrier |
|  | Neighbors of macrophage function |
|  | Neighbors of cellular senescence |
|  | Neighbors of colony formation |
|  | Neighbors of macrophage differentiation |
|  | Neighbors of cell differentiation |
|  | Neighbors of stem cell migration |
|  | Neighbors of life span |
|  | Neighbors of cell homeostasis |
|  | Neighbors of psoriasis |
|  | Neighbors of inflammatory disease |
|  | Neighbors of rheumatoid arthritis |
|  | Neighbors of atherosclerosis |
|  | Neighbors of fibrosis |
|  | Neighbors of colitis |
|  | Neighbors of arthritis |
|  | Neighbors of inflammatory bowel disease |
|  | Neighbors of injury |
|  | Neighbors of atherogenesis |
|  | Neighbors of vascular remodeling |
|  | Neighbors of metastasis |
|  | Neighbors of carcinogenesis |
|  | Neighbors of multiple sclerosis |
|  | Neighbors of chronic inflammation |
|  | Neighbors of inflammation |
|  | Neighbors of tumor microenvironment |
|  | Neighbors of pathological angiogenesis |
|  | Neighbors of obesity |
|  | Neighbors of vascular disease |
|  | Neighbors of diabetes mellitus |
|  | Neighbors of asthma |
|  | Neighbors of pneumonia |
|  | Neighbors of hyperplasia |
|  | Neighbors of osteoarthritis |
|  | Neighbors of liver fibrosis |
|  | Neighbors of chronic obstructive pulmonary disease |
|  | Neighbors of myocardial infarction |
|  | Neighbors of leukocyte infiltration |
|  | Neighbors of reperfusion injury |
|  | Neighbors of airway inflammation |
|  | Neighbors of hepatitis |
|  | Neighbors of insulin resistance |
|  | Neighbors of infection |
|  | Neighbors of vascular injury |
|  | Neighbors of renal injury |
|  | Neighbors of preeclampsia |
|  | Neighbors of macrophage infiltration |
|  | Neighbors of liver injury |
|  | Neighbors of neuroinflammation |
|  | Neighbors of sepsis |
|  | Neighbors of death |
|  | Neighbors of type 1 diabetes |
|  | Neighbors of encephalomyelitis |
|  | Neighbors of autoimmune disease |
|  | Neighbors of ischemic stroke |
|  | Neighbors of dermatitis |
|  | Neighbors of neutrophil infiltration |
|  | Neighbors of heart disease |
|  | Neighbors of diabetic nephropathy |
|  | Neighbors of lung injury |
|  | Neighbors of immunopathology |
|  | Neighbors of systemic sclerosis |
|  | Neighbors of hepatocellular carcinoma |
|  | Neighbors of autoimmunity |
|  | Neighbors of acute lung injury |
|  | Neighbors of gastric cancer |
|  | Neighbors of atherosclerotic plaque |
|  | Neighbors of experimental autoimmune encephalomyelitis |
|  | Neighbors of colorectal cancer |
|  | Neighbors of cardiac remodeling |
|  | Neighbors of breast cancer |
|  | Neighbors of ischemia |
|  | Neighbors of systemic lupus erythematosus |
|  | Neighbors of melanoma |
|  | Neighbors of collagen-induced arthritis |
|  | Neighbors of cartilage degeneration |
|  | Neighbors of interstitial fibrosis |
|  | Neighbors of neoplasm invasion |
|  | Neighbors of kidney disease |
|  | Neighbors of heart failure |
|  | Neighbors of lung disease |
|  | Neighbors of pulmonary fibrosis |
|  | Neighbors of cardiovascular disease |
|  | Neighbors of stroke |
|  | Neighbors of cancer |
|  | Neighbors of cerebral ischemia |
|  | Neighbors of type 2 diabetes |
|  | Neighbors of neoplasm |
|  | Neighbors of endometriosis |
|  | Neighbors of neointima |
|  | Neighbors of vasculitis |
|  | Neighbors of atrophy |
|  | Neighbors of respiratory hypersensitivity |
|  | Neighbors of myocardial fibrosis |
|  | Neighbors of coronary artery disease |
|  | Neighbors of rupture |
|  | Neighbors of endothelial cell dysfunction |
|  | Neighbors of liver disease |
|  | Neighbors of prostate cancer |
|  | Neighbors of hypertrophy |
|  | Neighbors of osteolysis |
|  | Neighbors of ovarian cancer |
|  | Neighbors of pancreatic cancer |
|  | Neighbors of hypertension |
|  | Neighbors of intimal hyperplasia |
|  | Neighbors of proteinuria |
|  | Neighbors of epithelial cell |
|  | Neighbors of keratinocyte |
|  | Neighbors of vascular smooth muscle cell |
|  | Neighbors of umbilical vein endothelial cell |
|  | Neighbors of monocyte |
|  | Neighbors of endometrial stromal cell |
|  | Neighbors of alveolar macrophage |
|  | Neighbors of synoviocyte |
|  | Neighbors of macrophage |
|  | Neighbors of adipocyte |
|  | Neighbors of activated monocyte |
|  | Neighbors of fibroblast |
|  | Neighbors of airway epithelial cell |
|  | Neighbors of PBMC |
|  | Neighbors of stromal cell |
|  | Neighbors of airway smooth muscle cell |
|  | Neighbors of osteoblast |
|  | Neighbors of pneumocyte |
|  | Neighbors of endometrium cell |
|  | Neighbors of microglia |
|  | Neighbors of trophoblast |
|  | Neighbors of intestine epithelium cell |
|  | Neighbors of mononuclear cell |
|  | Neighbors of hepatic stellate cell |
|  | Neighbors of hepatocyte |
|  | Neighbors of decidual cell |
|  | Neighbors of kidney mesangial cell |
|  | Neighbors of endothelial cell |
|  | Neighbors of dendritic cell |
|  | Neighbors of Kuppfer cell |
|  | Neighbors of proadipocyte |
|  | Neighbors of kidney tubule cell |
|  | Neighbors of inflammatory cell |
|  | Neighbors of bone marrow macrophage |
|  | Neighbors of peritoneal macrophage |
|  | Neighbors of osteoclast |
|  | Neighbors of neutrophil |
|  | Neighbors of smooth muscle myocyte |
|  | Neighbors of astrocyte |
|  | Neighbors of bone marrow derived dendritic cell |
|  | Neighbors of lung fibroblast |
|  | Neighbors of activated T-cell |
|  | Neighbors of mesothelial cell |
|  | Neighbors of mononuclear phagocyte |
|  | Neighbors of capillary endothelial cell |
|  | Neighbors of immunocompetent cell |
|  | Neighbors of granulocyte |
|  | Neighbors of cardiac myocyte |
|  | Neighbors of cancer-associated fibroblast |
|  | Neighbors of myeloid cell |
|  | Neighbors of mesenchymal stroma cell |
|  | Neighbors of corneal epithelial cell |
|  | Neighbors of cytotrophoblast |
|  | Neighbors of stem cell |
|  | Neighbors of tumor infiltrating macrophage |
|  | Neighbors of sinusoidal cell |
|  | Neighbors of B-cell |
|  | Neighbors of granulosa cell |
|  | Neighbors of leukocyte |
|  | Neighbors of chondrocyte |
|  | Neighbors of mesenchymal cell |
|  | Neighbors of T-cell |
|  | Neighbors of M1 macrophage |
|  | Neighbors of myofibroblast |
|  | Neighbors of pancreatic stellate cell |
|  | Neighbors of mast cell |
|  | Neighbors of bone marrow stromal cell |
|  | Neighbors of spleen cell |
|  | Neighbors of brain capillary endothelial cell |
|  | Neighbors of neuroglia |
|  | Neighbors of aortic smooth muscle cell |
|  | Neighbors of hematopoietic cell |
|  | Neighbors of adipose macrophage |
|  | Neighbors of podocyte |
|  | Neighbors of mammary epithelial cell |
|  | Neighbors of conventional dendritic cell |
|  | Neighbors of pericyte |
|  | Neighbors of antigen-presenting cell |
|  | Neighbors of epidermis cell |
|  | Neighbors of phagocyte |
|  | Neighbors of syncytiotrophoblast |
|  | Neighbors of lymphocyte |
|  | Neighbors of Langerhans cell |
|  | Neighbors of brain cell |
|  | Neighbors of M2 macrophage |
|  | Neighbors of lymphoid cell |
|  | Neighbors of retina cell |
|  | Neighbors of blood cell |
|  | Neighbors of skin cell |
|  | Neighbors of luteal cell |
|  | Neighbors of peripheral lymphocyte |
|  | Neighbors of activated B-cell |
|  | Neighbors of lung |
|  | Neighbors of liver |
|  | Neighbors of kidney |
|  | Neighbors of skin |
|  | Neighbors of intestine |
|  | Neighbors of lymph node |
|  | Neighbors of colon |
|  | Neighbors of airway |
|  | Neighbors of blood vessel |
|  | Neighbors of heart |
|  | Neighbors of aorta |
|  | Neighbors of microvessel |
|  | Neighbors of bone |
|  | Neighbors of placenta |
|  | Neighbors of carotid artery |
|  | Neighbors of synovial membrane |
|  | Neighbors of uterus |
|  | Neighbors of esophagus |
|  | Neighbors of periodontium |
|  | Neighbors of retina |
|  | Neighbors of brain |
|  | Neighbors of ovary |
|  | Neighbors of breast |
|  | Neighbors of blood vessel wall |
|  | Neighbors of stomach |
|  | Neighbors of islets of Langerhans |
|  | Neighbors of artery |
|  | Neighbors of cardiovascular system |
|  | Neighbors of central nervous system |
|  | Neighbors of kidney glomerulus |
|  | Neighbors of colorectal region |
|  | Neighbors of decidua |
|  | Neighbors of amnion |
|  | Neighbors of immune system |
|  | Neighbors of spleen |
|  | Neighbors of peritoneum |
|  | Neighbors of allantois |
|  | Neighbors of alveolus |
|  | Neighbors of coronary artery |
|  | Neighbors of left ventricle |
|  | Neighbors of uterine cervix |
|  | Neighbors of vein |
|  | Neighbors of arteriole |
|  | Neighbors of villus |
|  | Neighbors of bronchus |
|  | Neighbors of glands |
|  | Neighbors of nose |
|  | Neighbors of eye |
|  | Neighbors of cornea |
|  | Neighbors of hippocampus |
|  | Neighbors of intervertebral disc |
|  | Neighbors of vagina |
|  | Neighbors of vitreous body |
|  | Neighbors of cerebral cortex |
|  | Neighbors of brain blood vessel |
|  | Neighbors of myelin sheath |
|  | Neighbors of nervous system |
|  | Neighbors of periodontal ligament |
|  | Neighbors of trachea |
|  | Neighbors of corpus luteum of ovary |
|  | Neighbors of lower extremity |
|  | Neighbors of amniotic cavity |
|  | Neighbors of lymphatic vessels |
|  | Neighbors of left atrium |
|  | Neighbors of artery wall |
|  | Neighbors of NF-kB family |
|  | Neighbors of oxidized LDL |
|  | Neighbors of JNK |
|  | Neighbors of IL1 family |
|  | Neighbors of PPAR |
|  | Neighbors of Jun/Fos |
|  | Neighbors of NFKBI |
|  | Neighbors of PKC |
|  | Neighbors of PI3K |
|  | Neighbors of STAT family |
|  | Neighbors of mitogen-activated protein kinase |
|  | Neighbors of IFNAR ligand |
|  | Neighbors of transforming growth factor |
|  | Neighbors of TLR |
|  | Neighbors of cytokine |
|  | Neighbors of interferon |
|  | Neighbors of interleukin |
|  | Neighbors of JAK |
|  | Neighbors of MEK1/2 |
|  | Neighbors of PDGF |
|  | Neighbors of Ras GTPase |
|  | Neighbors of ERK1/2 |
|  | Neighbors of growth factor |
|  | Neighbors of PKA |
|  | Neighbors of ovalbumin |
|  | Neighbors of LDL |
|  | Neighbors of NF-AT family |
|  | Neighbors of beta adrenoceptor |
|  | Neighbors of chorionic gonadotropin |
|  | Neighbors of IKBK |
|  | Neighbors of protein tyrosine kinase |
|  | Neighbors of NAD(P)H oxidase |
|  | Neighbors of IgG |
|  | Neighbors of SMAD subfamily |
|  | Neighbors of AMPK |
|  | Neighbors of LXR |
|  | Neighbors of HDL |
|  | Neighbors of p38 |
|  | Neighbors of inflammatory cytokine |
|  | Neighbors of IL23 |
|  | Neighbors of estrogen receptor |
|  | Neighbors of T-cell receptor |
|  | Neighbors of histone deacetylase |
|  | Neighbors of PDGF-BB |
|  | Neighbors of IL1R |
|  | Neighbors of HIF-1 |
|  | Neighbors of GPCR |
|  | Neighbors of IL12 |
|  | Neighbors of Notch |
|  | Neighbors of prostaglandin-endoperoxide synthase |
|  | Neighbors of heat shock protein 90 |
|  | Neighbors of lectin |
|  | Neighbors of mitogen-activated protein kinase kinase |
|  | Neighbors of interleukin-35 |
|  | Neighbors of TSH |
|  | Neighbors of endothelin |
|  | Neighbors of NOS |
|  | Neighbors of superoxide dismutase |
|  | Neighbors of proteasome endopeptidase complex |
|  | Neighbors of class nuclear receptor with C4 zinc fingers |
|  | Neighbors of non-coding RNA |
|  | Neighbors of histone H3 |
|  | Neighbors of STAT5 |
|  | Neighbors of alpha2 adrenoceptor |
|  | Neighbors of vitronectin receptor (integrin) |
|  | Neighbors of PP2A |
|  | Neighbors of retinoid-X receptor subfamily |
|  | Neighbors of SAPK |
|  | Neighbors of WNT |
|  | Neighbors of immunoglobulin |
|  | Neighbors of phospholipase |
|  | Neighbors of AHR subfamily |
|  | Neighbors of ITG |
|  | Neighbors of relaxin |
|  | Neighbors of FSH |
|  | Neighbors of TORC1 |
|  | Neighbors of RA receptor |
|  | Neighbors of histone |
|  | Neighbors of Rho kinase |
|  | Neighbors of PDGF receptor |
|  | Neighbors of neuropeptides |
|  | Neighbors of pancreatic elastase |
|  | Neighbors of histone acetyltransferase |
|  | Neighbors of adrenoceptor |
|  | Neighbors of caspase |
|  | Neighbors of phosphoinositide phospholipase C |
|  | Neighbors of deacetylase |
|  | comon ps\_pos, pprarg\_neg targets |
|  | sars-cov no binding |
|  | Overview. Diseases of the digestive system (chapter 10) |
|  | Overview. Diseases of the respiratory (chapter 9) |
|  | 1\_Inflammation in keratinocytes in the pathology of acne |
|  | 2\_Interleukin-17 and interleukin-22 signaling in psoriasis |
|  | 1\_Osteoclast functional impairment in osteopetrosis |
|  | 3\_The role of interstitial fibroblasts in pyelonephritis |
|  | 3\_Mesangial cell dysfunction in glomerulonephritis |
|  | 2\_Cardiomyocyte hypertrophy |
|  | 2\_The intense proliferation and contractile activity of smooth muscle cells in pulmonary hypertension |
|  | 3\_Role of alveolar macrophages in pulmonary tissue destruction in COPD |
|  | 2\_3\_Eosinophilia and neutrophilia in asthma: Neutrophil chemotaxis and activation |
|  | 1\_Intestine epithelial cell dysfunction in ulcerative colitis |
|  | 1\_cd\_Amyloid-beta in Alzheimer's disease |
|  | drug-target moltransport |
|  | Interleukin-17 and interleukin-22 signaling in psoriasis |

---

|  |  |
| --- | --- |
| Group | Genes with Polymorphisms Associated with Migraine |
|  | Genes with Mutations Associated with Multiple Sclerosis |
|  | other partners for SARS |

---

|  |  |
| --- | --- |
| MedScan ID | 4318 |

---

|  |  |
| --- | --- |
| LocusLink ID | 4318 |
|  | 17395 |
|  | 81687 |

---

|  |  |
| --- | --- |
| Alias | Gel B |
|  | 92kD gelatinase |
|  | macrophage gelatinase |
|  | matrix metalloprotease 9 |
|  | 92-kDa type IV collagenases |
|  | 95 kDa type gelatinases |
|  | matrix metalloproteinase 9 |
|  | collagenase type IVB |
|  | 92kDa collagenase IV |
|  | 95 kDa type IV collagenase/gelatinase |
|  | matrix metallopeptidase 9 |
|  | collagenase type V |
|  | 92kDa gelatinase |
|  | MMP9 |
|  | GelB |
|  | 92kD type IV collagenase |
|  | collagenase type 5 |
|  | MANDP2 |
|  | AW743869 |
|  | type V collagenases |
|  | OTTHUMP00000031674 |
|  | pro-gelatinase B |
|  | proMMP9 |
|  | pro-MMP9 |
|  | type V collagenase |
|  | 92kDa type IV collagenase |
|  | RP23-61O3.10-001 |
|  | gelatinase B |
|  | progelatinase B |
|  | 3.4.24.35 |
|  | 92-kDa gelatinases |
|  | OTTMUSP00000001145 |
|  | CLG4B |
|  | macrophage gelatinases |
|  | RP23-61O3.10 |
|  | MMP-9 |
|  | matrix metallopeptidase 9 (gelatinase B, 92kDa gelatinase, 92kDa type IV collagenase) |
|  | matrix metalloproteinase 9 (gelatinase B, 92kDa gelatinase, 92kDa type IV collagenase) |
|  | matrix metalloproteinase-9 |
|  | B/MMP9 |
|  | pro-MMP-9 |
|  | 92 kDa gelatinase |
|  | 92 kDa type IV collagenase |
|  | 92-kDa type IV collagenase |
|  | matrix metalloproteinase 9 (gelatinase B, 92-kDa type IV collagenase) |
|  | matrix metalloproteinase 9 (gelatinase B 92-kDa type IV collagenase) |
|  | matrix metalloproteinase 9 (gelatinase b) |
|  | matrix metalloprotease 9 (gelatinase B, 92kDa gelatinase, 92kDa type IV collagenase) |
|  | matrix metalloproteinase 9 (gelatinase B, 92kD gelatinase, 92kD type IV collagenase) |
|  | matrix metalloprotease 9 (gelatinase b) |
|  | matrix metalloprotease 9 (gelatinase B, 92kD gelatinase, 92kD type IV collagenase) |
|  | matrix metalloprotease 9 (gelatinase B 92-kDa type IV collagenase) |
|  | 2 kDa 9 type IV collagenase |
|  | 92 kDa gelatinase |
|  | matrix metalloproteinase 9 preproprotein |
|  | B/MMP9s |

---

|  |  |
| --- | --- |
| EC Number | 3.4.24.35 |

---

|  |  |
| --- | --- |
| GO ID | 0005518 |
|  | 0004175 |
|  | 0004222 |
|  | 0008237 |
|  | 0004252 |
|  | 0008270 |
|  | 0071276 |
|  | 0034614 |
|  | 0030574 |
|  | 0019221 |
|  | 0007566 |
|  | 0035987 |
|  | 0048013 |
|  | 0022617 |
|  | 0030198 |
|  | 0030225 |
|  | 0043066 |
|  | 2001258 |
|  | 2001268 |
|  | 2000697 |
|  | 2001243 |
|  | 0043312 |
|  | 0043388 |
|  | 0030335 |
|  | 0045742 |
|  | 0051549 |
|  | 0001934 |
|  | 1900122 |
|  | 0090200 |
|  | 1904707 |
|  | 0006508 |
|  | 0150077 |
|  | 1904645 |
|  | 0001501 |
|  | 0062023 |
|  | 0070062 |
|  | 0005576 |
|  | 0005615 |
|  | 1904813 |
|  | 1904724 |
|  | 0001968 |
|  | 0016787 |
|  | 0046872 |
|  | 0008233 |
|  | 0044877 |
|  | 0007568 |
|  | 0071460 |
|  | 0007507 |
|  | 0048147 |
|  | 0045766 |
|  | 0043065 |
|  | 0002687 |
|  | 0031915 |
|  | 0051259 |
|  | 0042493 |
|  | 0006979 |
|  | 0048771 |
|  | 0019087 |
|  | 0031012 |
|  | 0032991 |
|  | 0071345 |
|  | 0071347 |
|  | 0071283 |
|  | 0071222 |
|  | 0071404 |
|  | 0071356 |
|  | 0001822 |
|  | 0050900 |
|  | 0001503 |
|  | 0007567 |
|  | 0032355 |
|  | 0045471 |
|  | 0009408 |
|  | 0009644 |
|  | 0055093 |
|  | 0001666 |
|  | 0032496 |
|  | 0009612 |
|  | 0035094 |
|  | 0007584 |
|  | 0014070 |
|  | 0010033 |
|  | 0009314 |
|  | 0032526 |
|  | 0034612 |
|  | 0033189 |
|  | 0005578 |
|  | 0032403 |
|  | 0043234 |
|  | 0007411 |
|  | 0060033 |
|  | 0016049 |
|  | 0008152 |
|  | 0005509 |
|  | 0005515 |

---

|  |  |
| --- | --- |
| KEGG ID | hsa:4318 |
|  | mmu:17395 |
|  | rno:81687 |

---

|  |  |
| --- | --- |
| Organism | Homo sapiens {Organism urn:agi-taxid:9606} |
|  | Mus musculus {Organism urn:agi-taxid:10090} |
|  | Rattus norvegicus {Organism urn:agi-taxid:10116} |
|  | Homo sapiens |
|  | Mus musculus |
|  | Rattus norvegicus |

---

|  |  |
| --- | --- |
| Mouse chromosome position | 2 85.27 cM |
|  | 2 96.0 cM |

---

|  |  |
| --- | --- |
| OMIM ID | 120361 |
|  | 613073 |
|  | 603932 |

---

|  |  |
| --- | --- |
| Rat chromosome position | 3q42 |

---

|  |  |
| --- | --- |
| Hugo ID | 7176 |
|  | HGNC:7176 |

---

|  |  |
| --- | --- |
| Human chromosome position | 20q13.12 |
|  | 20q11.2-q13.1 |

---

|  |  |
| --- | --- |
| Swiss-Prot Accession | P14780 |
|  | P14780.3 |
|  | P41245 |
|  | P41245.2 |
|  | P50282 |
|  | P50282.1 |
|  | B2R7V9 |
|  | Q3LR70 |
|  | Q8N725 |
|  | Q9H4Z1 |
|  | Q9UCJ9 |
|  | Q9UCL1 |
|  | Q9UDK2 |
|  | Q06788 |
|  | Q80XI8 |
|  | Q9DC02 |
|  | Q9Y354 |
|  | Q2M4J5 |
|  | Q3TTU7 |
|  | Q3UQ52 |
|  | Q6GXA2 |
|  | Q91ZS5 |
|  | Q9QWS6 |

---

|  |  |
| --- | --- |
| PIR ID | A34458 |
|  | I52580 |
|  | JC1456 |
|  | JC4364 |
|  | S62907 |

---

|  |  |
| --- | --- |
| GenBank ID | NC\_000020 |
|  | NM\_004994 |
|  | NP\_004985 |
|  | NG\_011468 |
|  | AF148064 |
|  | AAD37404 |
|  | AF538844 |
|  | AAM97934 |
|  | AL162458 |
|  | AX011001 |
|  | CAC07541 |
|  | CH471077 |
|  | EAW75776 |
|  | D10051 |
|  | BAA20967 |
|  | DQ194553 |
|  | ABA03169 |
|  | S83357 |
|  | AK298246 |
|  | BAH12743 |
|  | AK301446 |
|  | BAH13483 |
|  | AK302530 |
|  | BAH13736 |
|  | AK303080 |
|  | BAH13895 |
|  | AK311648 |
|  | AK313137 |
|  | BAG35956 |
|  | AK316145 |
|  | BAH14516 |
|  | AW468235 |
|  | BC006093 |
|  | AAH06093 |
|  | CN288579 |
|  | HQ874462 |
|  | AEA49029 |
|  | J05070 |
|  | AAA51539 |
|  | P14780 |
|  | NC\_000068 |
|  | XM\_006498861 |
|  | XP\_006498924 |
|  | NM\_013599 |
|  | NP\_038627 |
|  | AF000957 |
|  | AAC53571 |
|  | AF403768 |
|  | AAK97498 |
|  | AJ010318 |
|  | CAA09087 |
|  | AL591495 |
|  | AY902320 |
|  | AAX90605 |
|  | CH466551 |
|  | EDL06438 |
|  | D15060 |
|  | BAA03653 |
|  | X72794 |
|  | CAA51314 |
|  | AK004651 |
|  | BAB23442 |
|  | AK142787 |
|  | BAE25191 |
|  | AK159292 |
|  | BAE34968 |
|  | AK161176 |
|  | BAE36227 |
|  | AY622969 |
|  | AAT46400 |
|  | BC046991 |
|  | AAH46991 |
|  | BX524519 |
|  | D12712 |
|  | BAA02208 |
|  | S67830 |
|  | AAB28942 |
|  | X72795 |
|  | CAA51315 |
|  | Z27231 |
|  | CAA81745 |
|  | P41245 |
|  | NC\_005102 |
|  | NM\_031055 |
|  | NP\_112317 |
|  | AC\_000071 |
|  | AABR07054570 |
|  | AAHX01026383 |
|  | AJ438266 |
|  | CAD27893 |
|  | CH474005 |
|  | EDL96479 |
|  | U24441 |
|  | AAA90911 |
|  | U36476 |
|  | AAB01721 |
|  | P50282 |
|  | NC\_018931 |
|  | AMYH02037155 |
|  | AMYH02037156 |
|  | M68351 |
|  | M68355 |
|  | DQ896791 |
|  | ABM87790 |
|  | EU176415 |
|  | ABW03866 |
|  | AC\_000024 |
|  | AAHY01012603 |
|  | AC\_000152 |
|  | ABBA01052673 |
|  | ABBA01052674 |
|  | CAC10459 |
|  | CAM26464 |
|  | CAM26465 |
|  | NT\_011362 |
|  | NW\_927339 |
|  | NW\_001838666 |
|  | AC\_000063 |
|  | Q9Y354 |
|  | NT\_039207 |
|  | NW\_001030712 |
|  | Q2M4J5 |
|  | Q3TTU7 |
|  | Q3UQ52 |
|  | Q6GXA2 |
|  | Q80XI8 |
|  | Q91ZS5 |
|  | Q9QWS6 |
|  | NW\_001084817 |
|  | NW\_047660 |
|  | Q9UCJ9 |

---

|  |  |
| --- | --- |
| Swiss-Prot ID | MMP9\_HUMAN |
|  | MMP9\_MOUSE |
|  | MMP9\_RAT |
|  | Q80XI8\_MOUSE |

---

|  |  |
| --- | --- |
| Cell Localization | Secreted |
|  | Extracellular space |
|  | Extracellular matrix |
|  | Extracellular |
|  | Secreted, extracellular space, extracellular matrix |

---

|  |  |
| --- | --- |
| Ensembl ID | ENSG00000100985 |
|  | ENSP00000361405.3 |
|  | ENST00000372330.3 |
|  | ENSMUSG00000017737 |
|  | ENSMUSP00000017881.2 |
|  | ENSMUST00000017881.2 |
|  | ENSRNOG00000017539 |
|  | ENSRNOP00000023965.1 |
|  | ENSRNOT00000023965.3 |
|  | ENSP00000361405 |
|  | ENST00000372330 |
|  | ENSMUSP00000017881 |
|  | ENSMUST00000017881 |
|  | ENSRNOP00000023965 |
|  | ENSRNOT00000023965 |

---

|  |  |
| --- | --- |
| MGI ID | MGI:97011 |
|  | 97011 |

---

|  |  |
| --- | --- |
| RGD ID | 621320 |

---

|  |  |
| --- | --- |
| Unigene ID | Hs.297413 |
|  | Mm.4406 |
|  | Rn.10209 |

---

|  |  |
| --- | --- |
| Homologene ID | 3659 |

---

|  |  |
| --- | --- |
| IPI ID | IPI00027509 |
|  | IPI00196591 |
|  | IPI00319200 |
|  | IPI00330733 |
|  | IPI00751342 |

---
